# Author Correction: Mapping road network communities for guiding disease surveillance and control strategies

**DOI:** 10.1038/s41598-018-29782-z

**Published:** 2018-07-25

**Authors:** Emanuele Strano, Matheus P. Viana, Alessandro Sorichetta, Andrew J. Tatem

**Affiliations:** 1 Department of Civil and Environmental Engineering, Massachusetts Institute of Technology (MIT), Cambridge, MA 02139 USA; 2 German Aerospace Center (DLR), German Remote Sensing Data Center (DFD), Oberpfaffenhofen, D-82234 Wessling, Germany; 3 IBM Research Brazil, Sao Paulo, Brazil; 4 WorldPop, Department of Geography and Environment, University of Southampton, Highfield, Southampton UK; 5 Flowminder Foundation, Stockholm, Sweden

Correction to: *Scientific Reports* 10.1038/s41598-018-22969-4, published online 16 March 2018

This Article contains errors in the following equations:

In the Results section, under the subheading ‘Detecting communities in the African road network’, the equation:

$$\cos \,(\theta )={{\bf{r}}}_{i,j}{{\bf{r}}}_{j,p}/{{\bf{r}}}_{i,j}{{\bf{r}}}_{j,p},$$ where $${{\bf{r}}}_{i,j}={{\bf{r}}}_{j}{{\bf{r}}}_{i}$$

should read:

$$\cos \,(\theta )=|{{\bf{r}}}_{i,j}\cdot {{\bf{r}}}_{j,p}|/{{\rm{r}}}_{i,j}{{\rm{r}}}_{j,p},$$ where $${{\bf{r}}}_{i,j}={{\bf{r}}}_{j}-{{\bf{r}}}_{i}$$

In addition, under the same subheading, the equation:

$$P(k){k}^{-\gamma }$$

should read:

$$P(k)\sim {k}^{-\gamma }$$

Furthermore, Equation 1:

$${w}_{i,j}=\frac{1}{/{{\boldsymbol{r}}}_{i,j}/}{}_{{{\bf{r}}}_{i}\,}{}^{{{\bf{r}}}_{{{\rm{r}}}_{j}}}m({\bf{r}})p({\bf{r}})d({\bf{r}})$$

should read:

$${w}_{i,j}=\frac{1}{|{{\boldsymbol{r}}}_{i,j}|}{\int }_{{{\bf{r}}}_{i}}^{{{\bf{r}}}_{j}}m({\bf{r}})p({\bf{r}})d{\bf{r}}$$

